# Enrichment of Tc1 cells and T cell resistance to suppression are associated with dysglycemia in the visceral fat in human obesity

**DOI:** 10.1136/bmjdrc-2019-000772

**Published:** 2020-04-16

**Authors:** Sara Cardellini, Carlo Socci, Massimiliano Bissolati, Fioralba Pindozzi, Anna Giovenzana, Alessandro Saibene, Emanuele Bosi, Manuela Battaglia, Alessandra Petrelli

**Affiliations:** 1 San Raffaele Diabetes Research Institute, IRCCS Ospedale San Raffaele, Milano, Lombardia, Italy; 2 Transplant and Metabolic/Bariatric Surgery Unit, IRCCS Ospedale San Raffaele, Milano, Lombardia, Italy; 3 Department of General Medicine, Diabetes and Endocrinology, IRCCS Ospedale San Raffaele, Milano, Lombardia, Italy; 4 Vita-Salute San Raffaele University, Milan, Italy

**Keywords:** type 2 diabetes, obesity, T cells, visceral adipose tissue

## Abstract

**Objective:**

Insulin resistance, defined as tissue inflammation leading to type 2 diabetes, is a feature of obesity. The immune system has been implicated in its pathogenesis, but the role of adaptive immunity in humans remains uncertain. Here, we aim to determine whether specific phenotypic and functional properties of visceral adipose tissue (VAT)-derived CD4 conventional T cells (Tconv) and CD8 T cells are associated with dysglycemia in human obesity.

**Research design and methods:**

Peripheral blood and the stromal vascular fraction of obese patients without dysglycemia (n=23), with impaired fasting glucose or type 2 diabetes (n=17), and non-diabetic lean controls (n=11) were studied. Characterization of memory, activation profile, cytokine production, proliferative capacity, cytotoxic potential and transforming growth factor-β-mediated suppression of CD4 Tconv and CD8 T cells was performed. Correlation between anthropometric/metabolic parameters and VAT-derived T cell subsets was determined.

**Results:**

In the VAT of the overall obese population, reduced frequency of interferon-γ-producing or tumor necrosis factor-α-producing CD4 (ie, T helper 1, Th1) and CD8 (ie, cytotoxic type 1, Tc1) T cells, as well as interleukin-17-producing CD8 T cells (ie, Tc17), was evident when compared with lean controls. However, enrichment of Tc1 cells, together with the impaired ability of CD4 and CD8 T cells to be suppressed, distinguished the visceral fat of obese patients with dysglycemia from the one of non-diabetic obese patients. Moreover, accumulation of Th1 and Tc1 cells in the VAT correlated with anthropometric and metabolic parameters.

**Conclusions:**

Here, we define the VAT-specific characteristics of T cells in human obesity, showing that accumulation of Tc1 cells and T cell resistance to suppression can be harmful to the development of obesity-induced diabetes. These findings open new directions to investigate immunological targets in the obesity setting.

**Significance of this study**

**What is already known about this subject?**

- The immune system is involved in obesity-induced inflammation.
- Whether T cell subsets can modulate local obesity-induced diabetes in humans is still unknown.

**What are the new findings?**

- An overall impairment of T helper 1 (Th1), cytotoxic type 1 (Tc1) and Tc17 cells is evident in obese compared to lean visceral adipose tissue (VAT).
- When dysglycemia develops in obesity, accumulation of Tc1 cells as well as CD4 and CD8 T cell resistance to suppression occur in the VAT.

**How might these results change the focus of research or clinical practice?**

- Immunomodulatory approaches targeting T cells localized at the site of inflammation of obesity should be further investigated in obesity-induced type 2 diabetes.

## Introduction

Insulin resistance (IR) indicates the failure of cells to respond to insulin action and worsens when type 2 diabetes occurs, a condition commonly described as ‘diabesity’.^1^ Co-occurrence of obesity and type 2 diabetes is increasing, and available therapeutic approaches, such as insulin-sensitizing drugs or bariatric surgery, are only partially or temporarily efficacious (eg, 85% type 2 diabetes remission with bariatric surgery at 2 years drops to 50% at 5 years).^2 3^ A tight link between obesity, IR and type 2 diabetes is proven by the evidence that (1) obese subjects have higher chances of developing diabetes, (2) over 55% of obese patients have pre-diabetes or diabetes, and (3) weight gain/loss correlates with increasing/decreasing IR, respectively.^4 5^ Several factors, including genetic and epigenetic variants, as well as environmental components, have been associated with the development of type 2 diabetes.^6^ However, the reason why not all obese individuals develop diabetes still needs to be defined.^6^

Consistent pieces of evidence indicate that elevated levels of inflammatory molecules such as serum cytokines, chemokines and C reactive protein are present in the peripheral blood (PB) of obese patients, indicating that obesity is a systemic disease.^6^ However, key processes leading to IR occur in metabolically active organs, such as muscle and fat. IR, indeed, is triggered by low-level tissue chronic inflammation, induced by cytokine/chemokines and proinflammatory fatty acids-mediated mechanisms.^7^ Moreover, accumulation of ectopic lipid metabolites, endoplasmic reticulum stress, and immune pathways have all been implicated in the pathogenesis of IR.^6^

A growing literature focuses on the study of immunomodulatory activities of the adaptive immunity at target sites of inflammatory diseases, such as autoimmunity and cancer.^8 9^ Over the last few years, immunometabolism, which attempts to study the interface of immune and metabolic responses, became an attractive perspective in the field of obesity and metabolic disorders.^10^ The obesity setting differs from inflammatory environments such as autoimmunity, cancer and infections because the local inflammation is sterile and not driven by a cognate antigen. In obesity, the visceral adipose tissue (VAT), the accumulation of which associates with IR and type 2 diabetes,^11^ is featured by local infiltration of innate immune cells, such as proinflammatory macrophages (ie, M1).^12^ However, T cell subsets have also been associated with obesity-induced inflammation. Indeed, interferon (IFN)-γ-producing or tumor necrosis factor (TNF)-α-producing CD4 (ie, T helper 1, Th1) and CD8 (ie, cytotoxic type 1, Tc1) T cells, as well as interleukin (IL)-4-producing CD4 (ie, Th2) and CD8 (ie, cytotoxic type 2, Tc2) T cells, have been described to increase or decrease, respectively, in the VAT of obese mice.^13^ FoxP3+ regulatory T cells (Treg), instead, were found to be reduced in obesity.^14^ Unexpectedly, the VAT of obese mice was found enriched with CD8 T cells with a clonal T cell receptor (TCR) repertoire, suggesting local expansion toward cognate antigens with a potential implication of antigen specificity in obesity.^15^ T cells infiltrating the VAT have also been described in humans. Th1 and Tc1 cells were found increased,^16^ while impaired T cell regulation was observed in the VAT of obese patients^14^ when compared with subcutaneous adipose tissue (SAT) or with PB of the same patients. One report showed that IL-17-producing CD4 T cells (ie, Th17 cells), but not IL-17-producing CD8 T cells (ie, Tc17 cells), were increased in the VAT of obese patients with diabetes when compared with the VAT of both non-diabetic obese and lean subjects.^17^ However, most of the reports lack a proper control group, that is, the VAT of lean healthy donors, and rarely stratify the obese population in dysglycemic and normoglycemic subjects. Therefore, VAT-specific functional specialization of T cells in healthy subjects and obese patients with and without dysglycemia is still mostly unknown.

In this paper, we have characterized the phenotype and functional properties of VAT-derived CD4 conventional T cells (Tconv) and CD8 T cells in physiology (leanness condition) and in obesity with and without dysglycemia. Our results provide evidence of the crucial role of adaptive immunity in obesity-induced type 2 diabetes.

## Methods

### Subjects enrolled in the study

The subjects enrolled in this study underwent laparoscopic surgery at Ospedale San Raffaele, Milan (Italy) from February 2017 to November 2018. Three groups of individuals were enrolled in this study: morbidly obese patients (body mass index (BMI) ≥35 kg/m^2^) undergoing bariatric surgery (ie, sleeve gastrectomy), with either impaired fasting glucose or type 2 diabetes (OB-IFG&D); morbidly obese patients undergoing bariatric surgery without dysglycemia (OB-ND); and lean (BMI ≤25 kg/m^2^) non-diabetic controls (LC) undergoing kidney living donation. The following were the eligibility criteria for obese patients: (1) age 18–65; (2) candidates for bariatric surgery; (3) absence of morbid obesity severe complications; (4) absence of malignancies over the last 5 years; (5) absence of concomitant acute or chronic infections; and (6) absence of concomitant autoimmune or chronic inflammatory diseases. Criteria for the diagnosis of type 2 diabetes and impaired fasting glucose (IFG) were based on the American Diabetes Association guidelines.^18^ LC subjects were actively enrolled on the waiting list for kidney living donation and were free from hypertension, diabetes and chronic inflammatory diseases, including malignancies and infections.

### Data and sample collection

Medical history, current medications, anthropometric parameters (such as BMI, fat mass, waist to hip ratio and waist to height ratio), and blood tests, such as plasma glucose level, glycated hemoglobin (HbA1c), lipid profile, and liver and kidney function (estimated glomerular filtration rate calculated with the Chronic Kidney Disease Epidemiology Collaboration (CKD-EPI) equation), were obtained from all patients before surgery. Moreover, the Homeostasis Model Assessment of Insulin Resistance (HOMA2; measured with the calculator released by the Diabetes Trials Unit, University of Oxford), which accounts for variations in hepatic and peripheral glucose resistance,^19^ was assessed. PB and VAT, that is, omental visceral fat in case of obese patients and perirenal visceral fat in case of LC, were collected in the operatory room. PB was withdrawn before general anesthetic injection. The VAT was transferred to the laboratory in Dulbecco's Modified Eagle Medium (DMEM) supplemented with Bovine serum albumin (BSA), penicillin/streptomycin (P/S), and Hepes, and processed within 20 min from the collection.

### Isolation of PBMC and SVF

Peripheral blood mononuclear cells (PBMCs) were isolated by density gradient centrifugation using Lympholyte cell separation media (Cedarlane, cat# CL5020) and frozen in Roswell Park Memorial Institute (RPMI) supplemented with dimethyl sulfoxide (DMSO) (10%) and fetal bovine serum (20%) until further experimentation. The VAT was cut in small pieces, washed with PBS and incubated for 60 min at 37°C with Collagenase IV (Sigma, C5138) for enzymatic digestion. After filtering, a pellet of stromal vascular fraction (SVF) was obtained and counted.

### Fluorescence-activated cell sorter analysis

SVF (1×10^6^ cells) and whole blood (WB) were suspended in X-Vivo medium supplemented with human serum and P/S. Stainings were performed within 24 hours from the collection. For surface staining, they were incubated for 20 min in the dark with the following antihuman antibodies from BioLegend: anti-CD3 (Percp), anti-CD4 (BV711), anti-CD8 (BV605), anti-CD45RA (BV421), anti-CCR7 (PE) and anti-CD25 (APC-Cy7). The memory phenotype of CD4 Tconv and CD8 T cells was analyzed using CD45RA and CCR7, which allowed distinguishing naïve cells (T_N_), central memory cells (T_CM_), effector memory cells (T_EM_), and terminally differentiated cells (T_TEMRA_).^20^ Intracellular staining was performed using the eBioscience Foxp3/Transcription Factor Staining Buffer Set (ThermoFisher). After fixation and permeabilization, cells were incubated with anti-FoxP3 (Alexa 488, BioLegend), anti-Granzyme B (GzmB) (APC, Miltenyi Biotec) and anti-Ki-67 (PE-eFluor 610, eBioscience). For intracellular cytokine detection, SVF and WB were stimulated with phorbol myristate acetate (PMA) (10 ng/mL), ionomycin (500 ng/mL) and Brefeldin A (10 µg/mL) for 3 hours. After surface staining, fixation and permeabilization, the following antibodies were added: anti-FoxP3 (Alexa 488), anti-IFN-γ (Pacific Blue), anti-TNF-α (BV650) (BioLegend), anti-IL-10 (PE, BD Pharmingen) and anti-IL-17A (APC, eBioscience). Data were acquired on a BD LSRFortessa (BD Biosciences). Fluorescence intensity was standardized using multiple peak Rainbow Calibration Particles (Spherotech). Data were analyzed with FlowJo V.10.

### Proliferation and suppression assays

SVF and PBMC from obese patients and PBMC from LC were thawed, washed in RPMI, counted and incubated with carboxyfluorescein succinimidyl ester (CFSE) (CellTrace CFSE Cell Proliferation Kit, Life Technologies, ThermoFisher). After labeling, 300 000 cells were plated in round-bottom, 96-well plates coated with anti-CD3 (1 µg/mL, Miltenyi Biotec) to assess cell proliferation and cytokine production. In some conditions, cells were coincubated with transforming growth factor (TGF)-β (5 µg/mL, PeproTech) to assess T cell susceptibility to suppression. After 96 hours of incubation at 37°C, cells were stimulated with PMA/ionomycin following the protocol for intracellular cytokines detection. Per cent suppression was calculated using the following formula: ((% of proliferative T cells alone – % of proliferative T cells treated with TGF-β)/% of proliferative T cells alone) × 100.

### Statistical methods

Data are summarized as median (IQR) in tables and as mean±SE of measurement in graphs. Statistical analyses were conducted with GraphPad Prism V.7.00 software (GraphPad). When comparing two groups, data were analyzed using the Wilcoxon test when samples came from two different sites from the same subject (ie, PB vs VAT), or with the Mann-Whitney U test when samples came from two unrelated groups. In the case of comparison between nominal variables, Fisher’s exact test was used. When comparing more than two groups, Kruskal-Wallis test was used and corrected for multiple comparisons using the two-stage step-up method of Benjamini *et al.*^21^ To test the relationship between immune cell subsets and metabolic parameters, Spearman’s rank-order correlation analysis was performed. We rejected the null hypothesis when the p value was <0.05.

Before inclusion in the study, written informed consent was obtained from both the patients and the healthy donors.

## Results

### Patient characteristics

In this cross-sectional study, we aimed to address whether T cell phenotypic and functional characteristics in the visceral fat are associated with dysglycemia in obese patients. For this purpose, we have studied three groups of individuals: non-diabetic morbidly obese patients (OB-ND, n=23), dysglycemic morbidly obese patients with either IFG or type 2 diabetes (OB-IFG&D, n=17), and non-diabetic lean controls (LC, n=11). Given their homogeneity in terms of metabolic status (HOMA2: OB-IFG, 1.57 (1.07–3.57) vs OB-D, 2.38 (1.65–4.59), p=0.28), obese patients with IFG and type 2 diabetes were pulled together. As shown in table 1, LC were slightly older and prevalently female compared with the overall obese population, which is a consequence of the well-known elevated prevalence of female kidney living donors.^22^ Anthropometric parameters confirm the expected elevated BMI in both obese cohorts and show no difference in terms of BMI and fat mass percentage between OB-ND and OB-IFG&D, thus ruling out possible confounders that may interfere with the immunological asset.

**Table 1:** Patient characteristics

	OB-ND (n=23)	OB-IFG&D (n=17)	LC (n=11)	P value
Age (years)	42 (29–49)	47 (38–53)	51 (45–64)	(OB-ND vs LC)*
Sex (male/female)	5/18	11/6	1/11	(OB-ND vs OB-IFG&D)** (OB-IFG&D vs LC)**
BMI (kg/m^2^)	39.8 (37.0–44.0)	45.7 (40.6–49.4)	23.5 (20.3–25.6)	(OB-ND vs LC)*** (OB-IFG&D vs LC)***
Fat mass (%)	45.8 (49.6–43.2)	41.2 (38.0–47.9)	N/A	ns
Fasting glucose (mg/dL)	89.0 (81.8–92.3)	123.0 (110.5–156.0)	86.0 (72.0–90.0)	(OB-ND vs OB-IFG&D)*** (OB-IFG&D vs LC)***
HbA1c (%)	5.6 (5.3–5.8)	6.9 (6.1–7.5)	5.2 (5.1–5.3)	(OB-ND vs OB-IFG&D)*** (OB-IFG&D vs LC)***
HOMA2	1.37 (0.5–1.9)	1.95 (1.5–4.5)	0.45 (0.4–0.6)	(OB-IFG&D vs LC)*** (OB-ND vs LC)* (OB-ND vs OB-IFG&D)*
Total cholesterol (mg/dL)	185 (167.3–214.5)	185 (168.8–207.5)	204 (196.0–219.0)	ns
LDL-chol (mg/dL)	112.5 (96.5–130.0)	118.0 (99.0–127.5)	132.0 (102.5–136.0)	ns
HDL-chol (mg/dL)	50.5 (41.5–65.0)	38.0 (34.0–47.5)	64.0 (59.0–103.0)	(OB-ND vs OB-IFG&D)* (OB-IFG&D vs LC)***
TG (mg/dL)	107.5 (73.5–156.0)	145 (91.5–218.0)	83 (65.0–96.0)	(OB-IFG&D vs LC)**
ALT (U/L)	28.5 (17.8–43.0)	34.0 (22.0–51.5)	20.0 (14.8–22.3)	(OB-ND vs LC)* (OB-IFG&D vs LC)***
eGFR (mL/min/1.73 m^2^)	106.0 (93.9–122.0)	103.1 (96.4–111.3)	99.9 (86.8–104.1)	ns
Antidiabetic drugs	N/A	1/17 (insulin), 7/17 (metformin)	N/A	N/A

Values are shown as median (IQR).

The Kruskal-Wallis test corrected for multiple comparisons was used for all variables but sex. For sex, Fisher’s exact test was used.

*P<0.05, **P<0.01, ***P<0.001.

ALT, alanine aminotransferase; BMI, body mass index; eGFR, estimated glomerular filtration rate; Hb1Ac, glycated hemoglobin; HDL-chol, high-density lipoprotein cholesterol; HOMA2, Homeostasis Model Assessment of Insulin Resistance; LC, non-diabetic lean control; LDL-chol, low-density lipoprotein cholesterol; N/A, not available; OB-IFG&D, obese patients with impaired fasting glucose or type 2 diabetes; OB-ND, obese patient without dysglycemia; TG, triglycerides.

Moreover, metabolic status, assessed by fasting plasma glucose, HbA1c and HOMA2, was poor in OB-ND compared with LC and worsened further in obese patients with dysglycemia. Interestingly, total cholesterol and low-density lipoprotein cholesterol levels were similar between obese patients and LC, likely because 5 of 40 obese patients were undergoing lipid-lowering medications, whereas LC, who did not take medications, had a poor lipid profile. However, low levels of high-density lipoprotein (HDL) cholesterol and elevated triglycerides were observed in OB-IFG&D. Furthermore, while no differences in renal glomerular filtration were evident among groups, the liver enzyme alanine aminotransferase was found elevated in the overall obese population, indicating an initial liver damage.

### VAT from the overall obese population shows impaired Th1, Tc1 and Tc17 cells, whereas increased Tc1 cells distinguish patients with dysglycemia from non-diabetic obese

To assess the specific immune profile of T cells in the VAT in human obesity, we studied morbidly obese patients with and without dysglycemia and LC. A representative gating strategy used to distinguish T cell subsets in the VAT is shown in online supplementary figure S1. In this study, we focused on both CD4 Tconv, that is, non-activated CD25−FoxP3− CD4 T cells, and CD8 T cells. Here we aim to study a population of CD4 T cells as much homogeneous as possible; for this purpose, the following CD4 T cell subsets were excluded from the analysis: CD25+FoxP3+ Treg, CD25+FoxP3 cells, previously described as activated Tconv,^23^ and CD25−FoxP3+ cells, which were reported to be either activated Tconv^24^ or dysfunctional Treg.^25^

10.1136/bmjdrc-2019-000772.supp1 Supplementary data

**Figure 1:**
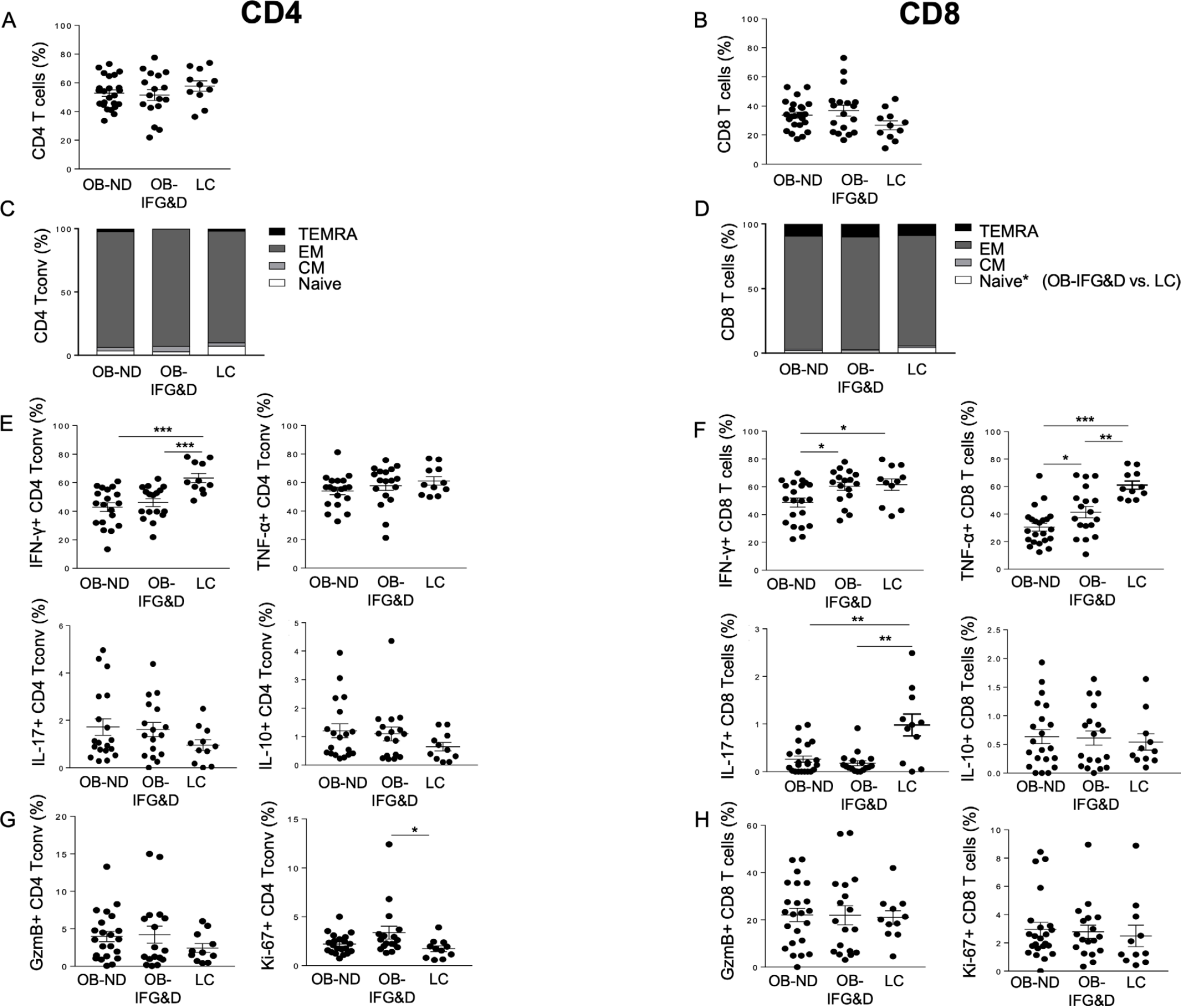
Distribution of CD4 Tconv and CD8 T cell subsets in the VAT of obese patients with and without dysglycemia. Frequency of (A–B) CD4 and CD8 T cells, (C–D) subsets of differentiation of CD4 Tconv and CD8 T cells based on CD45RA and CCR7 expression, (E–F) IFN-γ-producing and TNF-α-producing CD4 and CD8 T cells (ie, Th1 and Tc1 cells), IL-17-producing CD4 and CD8 T cells (ie, Th17 and Tc17), IL-10-producing CD4 and CD8 T cells, and (G–H) cytotoxic and proliferative cells was assessed in the VAT of the three groups studied. Kruskal-Wallis test corrected for multiple comparisons was used. *P<0.05, **P<0.01, ***P<0.001. CD45RA+CCR7+ (T_N_): naïve cells; CD45RA−CCR7+ (T_CM_): central memory cells; CD45RA−CCR7− (T_EM_): effector memory cells; CD45RA+CCR7− (T_TEMRA_): terminally differentiated cells. GzmB, Granzyme B; IFN-γ, interferon-γ; IL, interleukin; LC, non-diabetic lean control; OB-IFG&D, obese patients with impaired fasting glucose or type 2 diabetes; OB-ND, obese patients without dysglycemia; Tc1, cytotoxic type 1; Tconv, conventional T cells; Th1, T helper 1; TNF-α, tumor necrosis factor-α; VAT, visceral adipose tissue.

We first confirmed previous reports indicating that adipocyte morphology is altered in obesity regardless of the presence of diabetes.^26^ Indeed, the size of adipocytes in the VAT of obese patients was double compared with LC, with no differences observed between OB-ND and OB-D patients (data not shown). Increased frequency of total T cells was observed in the VAT of obese patients, regardless of the presence of diabetes, while no differences in the frequency of CD4 and CD8 T cells were evident (online supplementary figure S2 and figure 1A, B). In the VAT of the three groups studied, CD4 Tconv, CD25+FoxP3− and CD25−FoxP3+ CD4 T cells showed comparable frequency, while increased Treg frequency was observed in the VAT of OB-IFG&D patients compared with LC (online supplementary figure S3A–D). Although CD4 Tconv cell differentiation was comparable among the three groups, T_N_ CD8 T cells were found reduced in patients with dysglycemia compared with LC (figure 1C, D), and no altered distribution of memory subsets was observed. Unexpectedly, a reduction in IFN-γ-producing cells was observed in CD4 Tconv from both obese groups, while no differences were evident in the frequency of TNF-α, IL-17 and IL-10 cells (figure 1E).

Moreover, in the CD8 T cell compartment, an overall reduction of TNF-α-producing and IL-17-producing cells was evident in obese patients (figure 1F). These data indicate that Th1, Tc1 and Tc17 cells are reduced in obese VAT regardless of the presence of diabetes. However, both IFN-γ and TNF-α production by CD8 T cells were found increased in OB-IFG&D compared with OB-ND (figure 1F), indicating enrichment of Tc1 cells in obese patients with dysglycemia compared with non-diabetic obese. Furthermore, while the cytotoxic capacity was similar among the three groups, CD4 Tconv proliferation was found selectively increased in patients with dysglycemia compared with LC (figure 1G, H).

To conclude, the VAT of the overall obese population is characterized by a reduced representation of Th1, Tc1 and Tc17 cells. Remarkably, elevated frequency of Tc1 cells uniquely distinguishes the VAT of obese patients with dysglycemia from the one of non-diabetic obese.

### Enrichment of T cells with elevated proliferative capacity and decrease of cytotoxic CD8 T cells are obesity-specific features of VAT compared with PB

To further investigate whether the VAT of obese patients with and without dysglycemia is endowed with a peculiar T cell profile, we compared it with PB from the same individuals (table 2). Unlike LC, enrichment of total T cells was found in the VAT of obese patients regardless of the presence of diabetes. CD4 Tconv, instead, were found enriched in the VAT of LC, but not obese patients. In all the three groups studied, increased frequency of Th1 and Tc1 (ie, both IFN-γ-producing and TNF-α-producing CD4 Tconv and CD8 T cells) was found in the VAT, except for CD8 T cells from OB-ND patients. These data indicate that, already in physiological conditions, T cells producing proinflammatory cytokines enrich the VAT. Interestingly, reduced frequency of cytotoxic CD8 T cells was observed in the VAT of both OB-IFG&D and OB-ND patients. Reduction of cytotoxic CD8 T cells occurred only in OB-ND patients when considering the CD4 Tconv compartment. Finally, increased proliferation of both CD4 Tconv and CD8 T cells was observed in the VAT of both obese groups when compared with PB, indicating that VAT-specific cell activation occurs in the overall obese population.

**Table 2:** Frequency of T cell subsets in the PB and VAT of the three groups studied

Cell frequency (%)	OB-ND (n=23)			OB-IFG&D (n=17)			LC (n=11)		
	PB	VAT	P value	PB	VAT	P value	PB	VAT	P value
T cells	24.5 (15.7–26.7)	35.5 (28.9–47.4)	<0.0001	19.1 (17.1–30.9)	41.2 (32.9–56.3)	0.0005	25.3 (10.9–30)	22.4 (17.5–31.2)	ns
CD4 Tconv	92.1 (89.7–94)	93.9 (90–96.2)	ns	93.2 (88.9–96.2)	94.4 (90.8–97.2)	ns	94.5 (87.1–95.3)	97.4 (92.6–98.4)	0.009
IFN-γ+ CD4 Tconv	20.1 (9–36.2)	45.5 (31.9–55.1)	0.0002	18.9 (12.5–30.1)	51.6 (39.6–56.3)	<0.0001	15.4 (8.9–27.8)	60.3 (55.1–74.3)	0.001
TNF-α+ CD4 Tconv	32.6 (16.9–41.3)	56.2 (44.9–61.1)	0.0003	40.9 (25.0–45.6)	61.9 (55.8–68.8)	0.0008	29.2 (20–46.9)	58.3 (51.2–69.2)	0.001
IL-10+ CD4 Tconv	0.5 (0.4–0.8)	0.9 (0.4–1.9)	ns	0.6 (0.3–0.9)	1.0 (0.3–1.5)	ns	0.6 (0.3–1)	0.5 (0.2–1)	ns
IL-17+ CD4 Tconv	0.9 (0.7–1.3)	1.1 (0.7–3)	ns	0.8 (0.7–1.2)	1.4 (0.5–2.6)	ns	0.7 (0.3–1.2)	0.9 (0.2–1.6)	ns
GzmB+ CD4 Tconv	7.2 (2–10.9)	3.6 (1.3–6.1)	0.0009	4.5 (1.0–26.6)	2.1 (0.9–6.4)	ns	1.1 (0.3–3.2)	1.9 (0.7–4.3)	ns
Ki-67+ CD4 Tconv	1.7 (1.3–2.5)	2.2 (1.5–2.9)	0.008	1.4 (0.8–1.8)	2.7 (1.9–3.4)	0.01	1.4 (0.8–1.9)	1.7 (0.8–2.4)	ns
CD8 T cells	31.7 (24.9–36.1)	33.8 (27.1–40.3)	ns	26.5 (21.4–38.9)	40 (23.5–43.1)	ns	24.5 (20.2–28.9)	26.5 (18.8–32.9)	ns
IFN-γ+ CD8 T cells	38.6 (29.9–56.2)	50.5 (33.6–61.8)	ns	40.3 (24.9–60.3)	63.3 (52.5–69.6)	0.007	23.4 (20.1–39.2)	63.9 (44.8–75.3)	0.001
TNF-α+ CD8 T cells	23.1 (12.2–41.2)	28.4 (20.6–36.6)	ns	30.2 (11.3–45.4)	41.6 (31.7–55.9)	0.04	27.9 (11.6–40.3)	43.4 (27.9–58.5)	0.002
IL-10+ CD8 T cells	0.4 (0.2–0.7)	0.5 (0.2–1)	ns	0.3 (0.1–0.5)	0.7 (0.1–1.0)	ns	0.3 (0.3–0.9)	0.4 (0.2–0.7)	ns
IL-17+ CD8 T cells	0.2 (0.03–0.5)	0.2 (0–0.5)	ns	0.1 (0.0–0.2)	0.1 (0.0–0.2)	ns	0.1 (0.02–0.2)	0.2 (0–0.9)	ns
GzmB+ CD8 T cells	31.0 (17.8–51.8)	22.4 (9–31.7)	0.0002	40.4 (19.2–63.2)	19.5 (6.4–35.0)	0.002	15.9 (8.9–46.6)	20.2 (14.2–26.8)	ns
Ki-67+ CD8 T cells	1.2 (0.7–2.1)	2.3 (1.7–3.4)	<0.0001	1.3 (0.6–2.6)	2.4 (1.4–3.8)	0.02	1.6 (0.8–2.6)	1.3 (0.8–3.9)	ns

The Wilcoxon test was used for comparisons between PB and VAT of the same patient.

GzmB, Granzyme B; IFN-γ, interferon-γ; IL, interleukin; LC, non-diabetic lean controls; OB-IFG&D, obese patients with impaired fasting glucose and type 2 diabetes; OB-ND, obese patients without dysglycemia; PB, peripheral blood; Tconv, conventional T cells; TNF-α, tumor necrosis factor-α; VAT, visceral adipose tissue.

These data have shown that, in comparison with PB coming from the same individuals, enrichment of T cells, decrease of cytotoxic CD8 T cells, as well as elevated proliferative capacity of both CD4 Tconv and CD8 T cells are obesity-specific characteristics of the VAT, regardless of the presence of diabetes.

### Hyper-responsiveness of CD4 and CD8 T cells occurs in obese VAT compared with PB, but resistance to suppression occurs only in VAT of patients with diabetes

**Figure 2:**
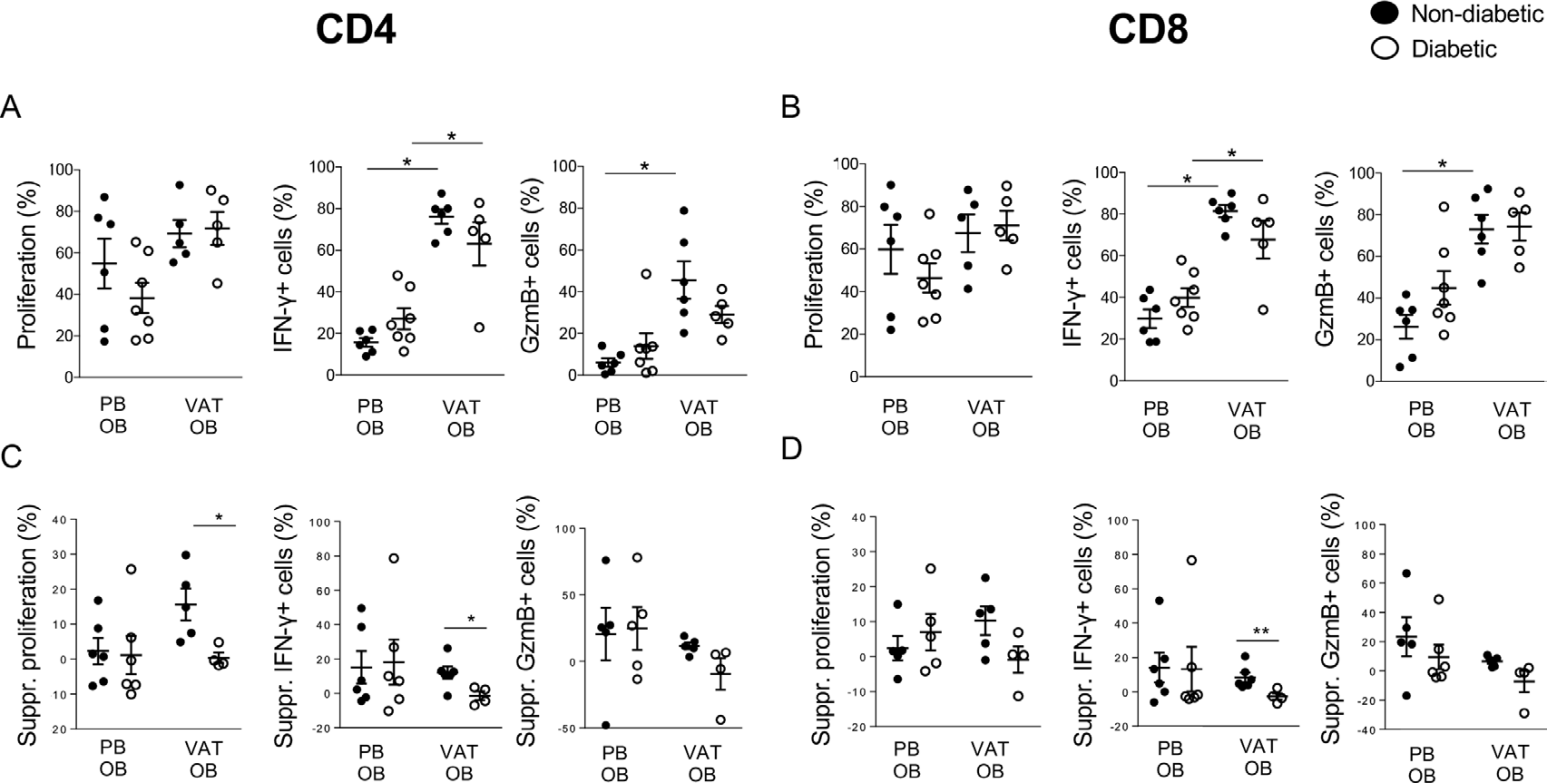
CD4 and CD8 T cell activation and cytokine-mediated suppression in the VAT of OB patients with and without type 2 diabetes. (A–B) Cell proliferation (measured by CFSE dilution), IFN-γ-producing and GzmB-producing cells were measured on CD4 and CD8 T cells on in vitro TCR stimulation of PBMC and VAT from OB patients with (empty circle) and without (full circle) type 2 diabetes. The Mann-Whitney U test was used for comparisons. (C–D) Percentage of suppression (suppr) of proliferation, IFN-γ-producing and GzmB-producing cells on CD4 and CD8 T cells on coincubation with the suppressive cytokine TGF-β in PBMC and VAT from OB patients with (empty circle) and without (full circle) type 2 diabetes. The Mann-Whitney U test was used for comparisons. *P<0.05, **P<0.01, ***P<0.001. CFSE, carboxyfluorescein succinimidyl ester; GzmB, Granzyme B; IFN-γ, interferon-γ; OB, obese; PB, peripheral blood; PBMC, peripheral blood mononuclear cells; TCR, T cell receptor; TGF-β, transforming growth factor-β; VAT, visceral adipose tissue.

To determine whether the inflammatory milieu present in obese VAT with dysglycemia affects the ability of T cells to be activated and functionally modulated, TCR stimulation and cytokine-mediated suppression assays were performed. CD4 and CD8 T cells from PB of the overall obese population (ie, non-diabetic obese and obese patients with type 2 diabetes) showed a similar level of proliferation/activation on in vitro stimulation compared with LC (data not shown). Interestingly, VAT-derived T cells from obese patients showed no differences in terms of proliferation, but higher production of IFN-γ when compared with PB of the same patient (figure 2A, B). Elevated GzmB production was evident in the VAT of OB-ND, with a trend observed in OB-D (p=0.06; figure 2A, B).

The susceptibility to cytokine-mediated suppression was assessed incubating samples from obese patients with and without type 2 diabetes with TGF-β. While in PB of obese patients no differences were observed between OB-ND and OB-D, impaired suppression of proliferation and IFN-γ production was evident in CD4 T cells from the VAT of patients with type 2 diabetes. In the CD8 T cell compartment, we also observed impaired suppression of IFN-γ production and a trend of impaired GzmB suppression (p=0.06) (figure 2C, D).

Overall, these data indicate that, compared with PB, VAT-derived CD4 and CD8 T cells display hyper-responsiveness in patients with obesity regardless of the presence of diabetes. Notably, resistance to cytokine-mediated suppression is a unique feature of the VAT from obese patients with dysglycemia.

### Frequency of VAT-derived Th1 and Tc1 cells correlates with anthropometric and metabolic parameters

**Figure 3:**
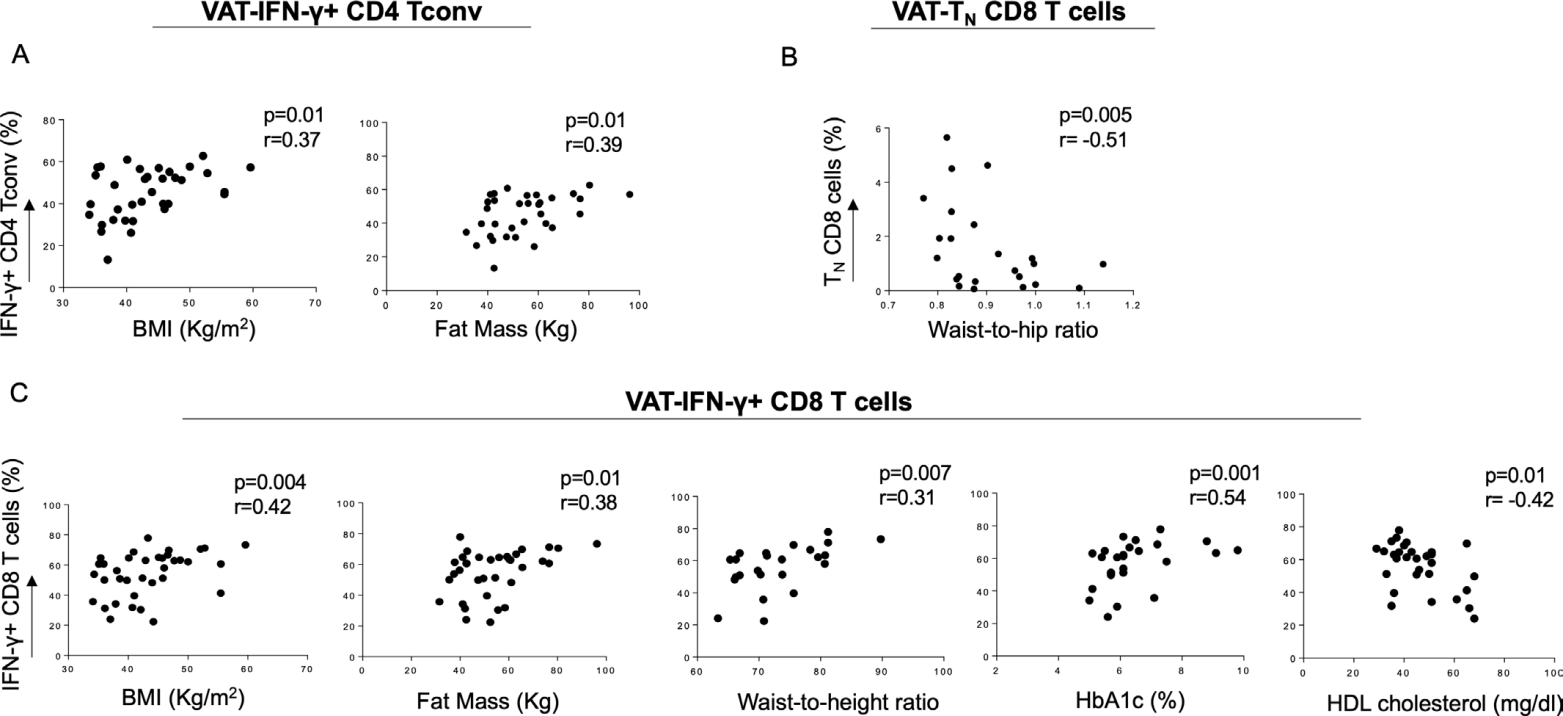
Correlation of anthropometric and metabolic parameters with PB and VAT-derived T cell subsets in obese patients. (A) BMI and fat mass directly correlated with VAT-derived IFN-γ-producing CD4 Tconv, while (B) T_N_ CD8 T cells showed a negative correlation with waist to hip ratio. (C) VAT-derived IFN-γ-producing CD8 T cells directly correlated with BMI, fat mass, waist to height ratio and HbA1c, while a negative correlation with HDL cholesterol was evident. Spearman’s rank-order test. BMI, body mass index; HbA1c, glycated hemoglobin; HDL, high-density lipoprotein; IFN-γ, interferon-γ; PB, peripheral blood; Tconv, conventional T cells; T_N_, naïve cells; VAT, visceral adipose tissue.

We then assessed whether the frequency of VAT-derived CD4 Tconv and CD8 T cell subsets analyzed in this study was associated with anthropometric and metabolic measurements.^27 28^ Despite the overall reduction of IFN-γ+ CD4 Tconv in obese patients, BMI and fat mass positively correlated with the frequency of this cell subset (figure 3A), indicating weight-dependent accumulation in the VAT. Moreover, T_N_ CD8 cells, which were reduced in the VAT of obese patients with dysglycemia, showed an inverse correlation with waist to hip ratio (figure 3B). Interestingly, VAT-derived IFN-γ-producing CD8 T cells positively correlate with BMI, fat mass and waist to height ratio, as well as glucose control, assessed by serum glucose (data not shown) and A1c levels (figure 3C). A negative correlation instead was evident with HDL cholesterol, known to be protective against cardiovascular diseases (figure 3C). No correlation between TNF-α-producing CD8 T cells infiltrating the VAT of obese subjects and anthropometric/metabolic parameters was observed (data not shown).

Overall, these data show that, in obese patients, anthropometric and metabolic parameters correlate with Th1 and Tc1 cells localized in the VAT, thus suggesting a possible association between accumulation of specific immune cells at the site of inflammation of obesity and the metabolic status.

## Discussion

Obesity and type 2 diabetes are considered epidemics of modern society and represent an economic issue due to elevated healthcare costs.^29^ Obesity-induced IR, together with pancreatic beta-cell failure, is a prerequisite to the development of type 2 diabetes.^6^ However, the mechanisms underlying the link between obesity and type 2 diabetes are still uncertain. Systematically comparing PB and VATs from obese patients with and without dysglycemia and non-diabetic LC, we have addressed phenotypic and functional alterations of CD4 Tconv and CD8 T cells at the obesity site of inflammation, thus elucidating the role of adaptive immunity in the development of obesity-induced type 2 diabetes.

Given the ability of proinflammatory cytokines such as TNF-α and IFN-γ to induce IR,^30 31^ it is expected that these cytokines are enriched at the site of inflammation of obesity (ie, the VAT). Here we showed that the enrichment of Th1 and Tc1 cells in the VAT compared with PB is not a specific feature of obesity and that it also occurs in diabetes-free lean subjects, indicating that this is instead a general feature of VAT. Unexpectedly, when comparing VATs, T cells from the overall obese population displayed reduced frequency of Th1, Tc1 and Tc17 cells compared with LC. In preclinical models, Th1 cells have been shown to accumulate in the VAT of obese mice.^13^ In humans, conflicting data have been reported, likely because comparisons are often performed with SAT or with PB rather than with VAT from lean donors.^16^ In a report comparing VATs, no enrichment of proinflammatory T cells was described in obesity.^32^ A recent publication also shows that increased Th17, but not Tc17, occurs in the VAT of obese patients compared with lean controls, with a further enrichment of this subset in the presence of type 2 diabetes.^17^ This report is not in line with our data showing a shortage of Tc17 cells in obesity. These conflicting data may be explained by the stringent selection criteria used for LC in our study, in which control subjects are a unique population of healthy individuals free from metabolic, cardiovascular, infectious and cancer diseases.

We also show that elevated Tc1 cells (ie, IFN-γ-producing and TNF-α-producing CD8 T cells) accumulated in the VAT of OB-IFG&D patients compared with OB-ND, suggesting that this may represent a mechanism contributing to local IR. Indeed, both cytokines have been described to reduce the phosphorylation of the Akt signaling and to induce IR in adipocytes.^30 31^ Moreover, anti-TNF therapy improved insulin sensitivity and reversed defects in the insulin signaling cascade in patients with rheumatic diseases.^33^ TNF-α and IFN-γ might participate in maintaining local homeostasis, as demonstrated by their elevated levels in the VAT of LC, thus being protective from weight gain. However, in the obese setting, shortage of Tc1 cells in the VAT is observed, which turns, once dysglycemia occurs, into Tc1 accumulation. Our results may indicate that IR and type 2 diabetes development may be exacerbated by the accumulation of TNF-α and IFN-γ in a deprived environment. Further investigation of the mechanisms underlying cytokine-mediated induction of IR by CD8 T cells is required.

Interestingly, the T cell infiltrate in the VAT of obese patients with IFG and type 2 diabetes was found superimposable, which prompted us to pool together these patients in one group. This evidence indicates that alterations of the immune infiltrate in the VAT occurs already at preclinical stages of diabetes and that they are likely not due to glucotoxicity.

At the site of inflammation of chronic inflammatory diseases, such as autoimmune arthritis or inflammatory bowel diseases, T cell activation is accompanied by resistance to suppression.^34^ Similarly, VAT-derived CD4 and CD8 T cells from obese patients are hyper-responsive when stimulated. Our data also show that lack of susceptibility to suppression is a type 2 diabetes-specific feature of VAT-derived CD4 and CD8 T cells. Therefore, despite the elevated levels of the suppressive cytokine TGF-β observed in obese VAT,^32^ once type 2 diabetes occurs, T cells are no longer susceptible to its regulatory action. This finding also explains why proinflammatory CD8 T cells and proliferative CD4 Tconv are elevated compared with the non-diabetic condition. It is likely that, in the obese setting, featured by altered adipocyte morphology and accumulation of inflammatory macrophages, dysglycemia occurs when proinflammatory T cells resistant to suppression are recruited. As previously shown by our group in an autoimmune setting,^35^ elevated TNF-α and IFN-γ production by T cells may be responsible for the T cell resistance to suppression via an autocrine mechanism.

A strength of this study is the systematic comparison between VAT from obese patients and LC. LC-derived VAT, obtained from a population of healthy subjects undergoing abdominal surgery for kidney donation, is the proper control for our scientific questions. However, due to differences in gender (ie, mainly female LC) and location of the visceral fat depot in obese and lean individuals (ie, omental vs perirenal, respectively), these results require further validation.

We observed that the frequency of VAT-derived Th1 and Tc1 cells correlates with anthropometric and metabolic parameters. This evidence further supports the idea that accumulation of T cell-derived proinflammatory cytokines in the VAT of obese patients may represent the link between obesity and IR and a risk factor for the development of type 2 diabetes. Given that anthropometric and metabolic parameters are predictors of cardiovascular diseases,^27 28^ it should be further investigated whether specific T cell subsets may be used as a biomarker of cardiovascular risk. Although a cause–effect relationship cannot be established between obesity-induced type 2 diabetes and T cells, our data suggest that metabolic disorders may have a detrimental effect on T cell differentiation and phenotype, which in turn may promote the development of IR and type 2 diabetes.

Even though the adaptive immune response appears to be relevant in the context of obesity, innate immunity displays a crucial role as well. Indeed, macrophages account for more than 50% of the immune cells infiltrating adipose tissue in obesity,^36^ and their accumulation in obese VAT is preceded by local enrichment of CD8 T cells.^12^ Macrophages secrete the majority of inflammatory cytokines,^37^ and they are polarized toward a unique proinflammatory phenotype, which can be induced by external stimuli (ie, glucose, insulin, and palmitate).^38^ Defining both macrophages and T cell dynamics of infiltration in time and space, as well as the assessment of their local interactions, is crucial to elucidate the immune mechanisms of IR.

In this paper, we show that obesity-specific as well as ‘diabesity’-specific features of T cells can be identified at the site of inflammation. The well-established notion that the visceral fat in obesity is enriched with proinflammatory molecules, at least in humans, does not apply to T cells. This is shown by the evidence that (1) enrichment of TNF-α-producing and IFN-γ-producing T cells compared with PB is not a specific feature of obesity, but it occurs in lean VAT too; and (2) a shortage of Th1, Tc1 and Tc17 cells is evident in obese VAT compared with the leanness condition. Nonetheless, when dysglycemia develops in the context of obesity, enrichment of Tc1 cells, accompanied by impaired susceptibility of T cells to suppression, occurs. These results provide evidence for the in vivo relevance of Tc1 cells in the visceral fat of obese patients with dysglycemia, showing that fine-tuning of CD8 T cell effector functions may be harmful to the development of obesity-induced type 2 diabetes. Our results open up new avenues to investigate how the modulation of adaptive immunity can be used for therapeutic intervention in the context of human obesity.
