# Supplementary figures and images for: Enrichment of Tc1 cells and T cell resistance to suppression are associated with dysglycemia in the visceral fat in human obesity
Source: BMJ Open Diabetes Res Care. 2020 Apr 16;8(1):e000772. doi: 10.1136/bmjdrc-2019-000772 (PMC7199176; doi:10.1136/bmjdrc-2019-000772)

Supplementary file

Supplementary Figures

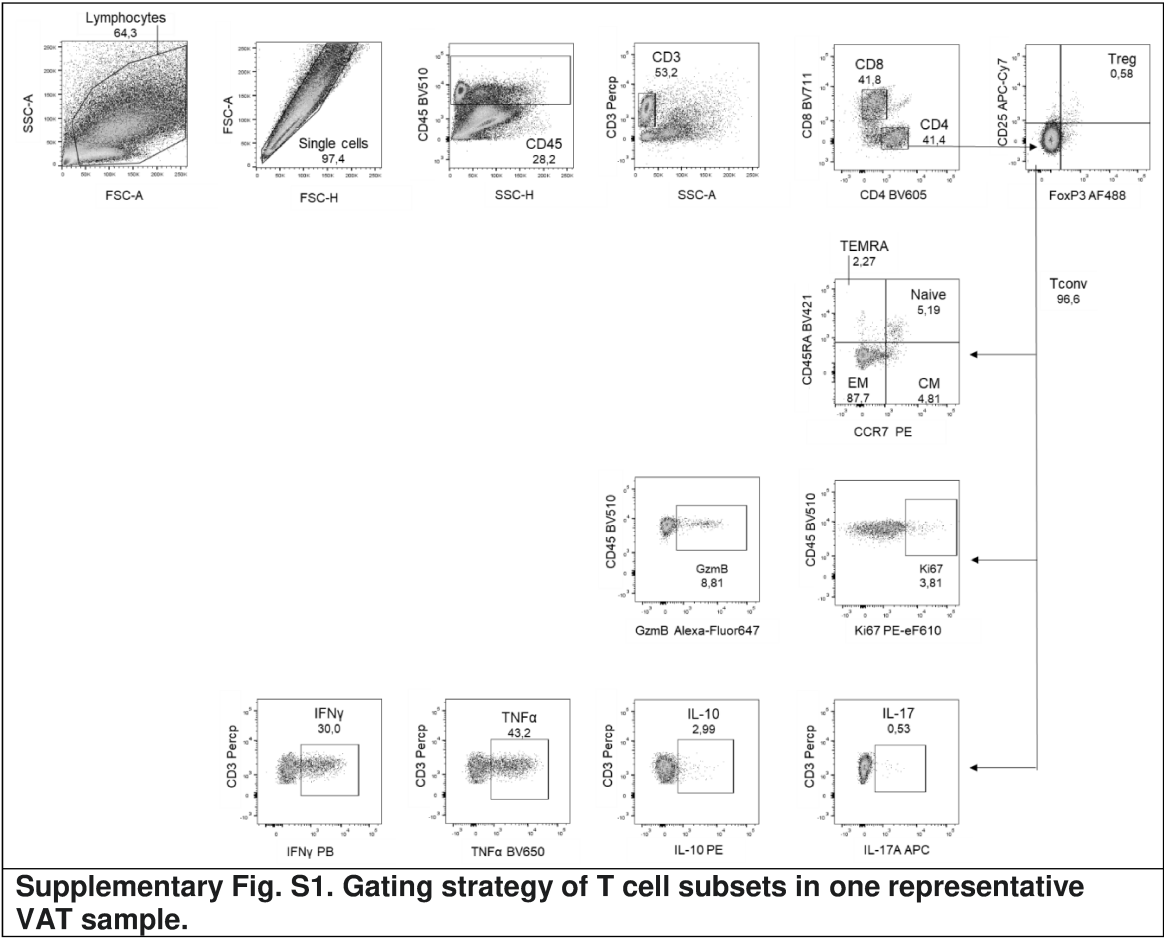

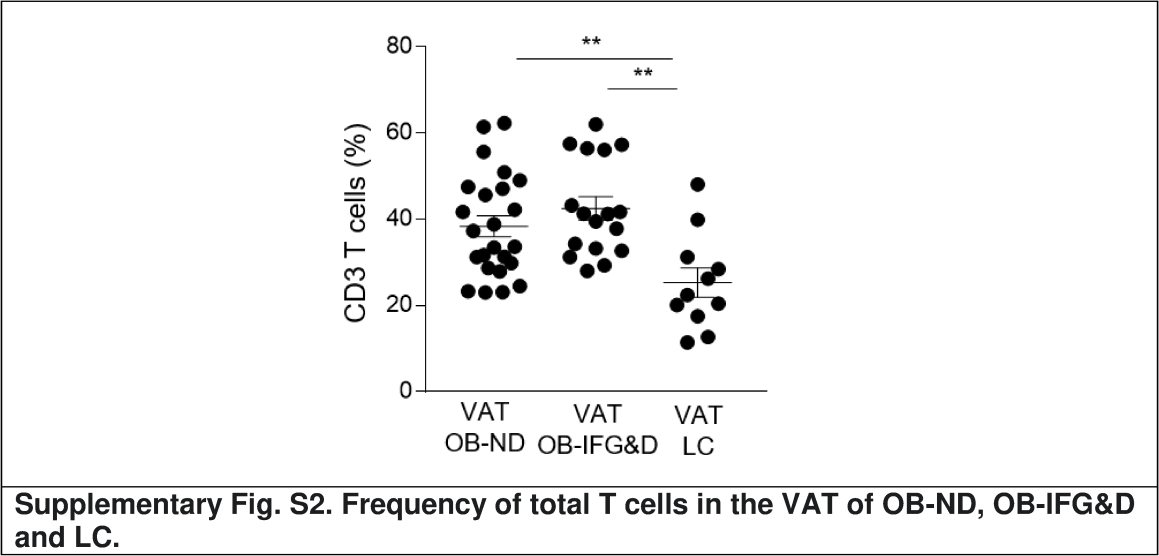

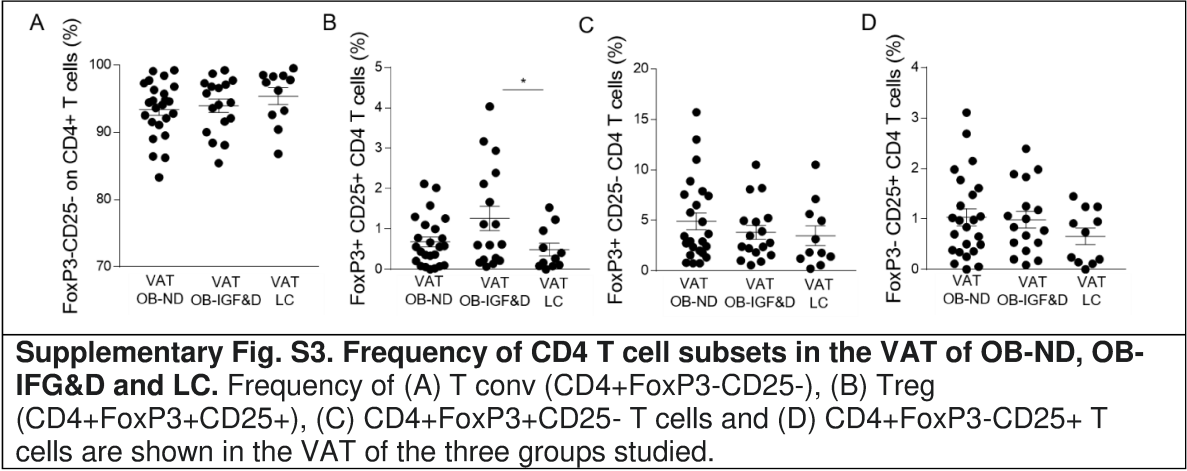

Supplement: Supplementary data [file bmjdrc-2019-000772supp001.pdf]
